# Anticancer Effect of *Enterococcus faecium*, Isolated from Vaginal Fluid, on Ovarian Cancer Cells

**DOI:** 10.61186/ibj.3846

**Published:** 2023-07-03

**Authors:** Soraya Pourmollaei, Azizeh Farshbaf-Khalili, Abolfazl Barzegari, Sepideh Bastani, Soraya Babaie, Amir Fattahi, Mahnaz Shahnazi

**Affiliations:** 1 Department of Midwifery, Faculty of Nursing and Midwifery, Tabriz University of Medical Sciences, Tabriz, Iran;; 2 Students' Research Committee, Tabriz University of Medical Sciences, Tabriz, Iran;; 3 Aging Research Institute, Physical Medicine, and Rehabilitation Research Centre, Tabriz University of Medical Sciences, Tabriz, Iran;; 4 Research Center of Pharmaceutical Nanotechnology, Biomedicine Institute, Tabriz University of Medical Sciences, Tabriz, Iran;; 5 Womenʼs Reproductive Health Research Center, Tabriz University of Medical Sciences, Tabriz, Iran

**Keywords:** Apoptosis, Enterococcus faecium, Ovarian neoplasms

## Abstract

ABSTRACT

**Background:**

Given the association between cervicovaginal microbiota and OVC, we investigated the effect of *E. faecium *CM on OVC (Caov-4) cells.

**Methods:**

CM was obtained from the bacterium *E. faecium *isolated from the vagina of healthy women. The Caov-4 cells were treated with varying concentrations of CM that comprised co-cultured bacteria with 0.2, 0.5, 1, 1.5, and 2 OD for 12, 24, and 48 h. The apoptosis and growth of cancer cells were evaluated by DAPI staining, flow cytometry, and DNA laddering assay. Moreover, the expression of *PTEN*, *BAX*, *BCL2*, and *AKT1* genes were analyzed using real-time PCR.

**Results:**

The CM at a concentration of 0.5 OD from the cultured bacteria and incubation time of 48 h showed the highest negative effect on the viability of cancer cells. The CM treatment increased DNA fragmentation and also induced apoptosis in Caov-4 cells. Interestingly, CM could decrease the expression of proapoptotic genes were less, while antiapoptotic genes were more than 5-FU in the presence of CM.

**Conclusion:**

CM of human-derived *E. faecium* could have an anticancer effect on OVC cells in a concentration- and time-dependent manner. This study demonstrated that *E. faecium* secretes anticancer substances into the CM, which could directly affect the viability and apoptosis of cancer cells.

## INTRODUCTION

Ovarian cancer is the second most prevalent cancer of the genital system and associated with the highest mortality rate among all female malignancies^[^^1^^]^. Statistics have shown that the chance of five-year survival is about 40% among OVC patients in Asia and Europe^[^^2^^]^. Diagnosis of OVC is relatively difficult due to the nonspecific symptoms of cancer. In this regard, it has been reported that around 80% of OVC cases are diagnosed at stage III or IV^[^^1^^,^^3^^]^. 

The standard first-line treatment for advanced epithelial OVC is maximal cytoreductive debulking surgery, followed by platinum-based chemotherapy. In addition to the high rate of remission in primary therapy, the incurable recurrent disease can be observed in approximately 85% of the OVC cases. Therefore, the identification of new therapeutic strategies for OVC seems necessary^[^^4^^]^. 

Previous research has demonstrated a correlation between OVC and its risk factors, specifically the presence of less than 50% lactobacilli in cervicovaginal microbiota^[^^5^^]^. Additional research has revealed that vaginal lactobacilli have the capability of impeding the proliferation of other pathogenic bacteria. However, alterations in the bacterial community are indicative of the likelihood of OVC development^[^^6^^,^^7^^]^. Hence, application of probiotics to regulate and stabilize the host microbiomes has been suggested as an attractive strategy for OVC treatment ^[^^8^^]^. It has been postulated that the anticancer properties of probiotics are due to their ability to regulate cell apoptosis via both intrinsic and extrinsic pathways^[^^8^^-^^11^^]^. However, not much is known about the anticancer effect of human-derived enterococcal strains on OVC. Recently, it has been reported that commensal *E. faecium* could suppress the proliferation of cervical, lung, and colon cancer cell lines^[^^12^^]^. 

Given the beneficial effects of probiotics on cancer and their potential for apoptosis induction, the current study investigated the ability of *E. faecium*, isolated from the vagina of healthy women, to inhibit the growth and induce the apoptosis of OVC (Caov-4) cells.

## MATERIALS AND METHODS

*Sampling and isolation of bacteria*

Bacterial specimens were obtained from the vagina of 10 healthy females referred to Al-Zahra Hospital (Tabriz, Iran). The samples were diluted with 500 μl of PBS. The diluted samples were cultured on MRS agar (Liofichem Bacteriology Products, Italy) and incubated at 37 °C for 48 h. Then the single colony created on MRS agar was transferred to 5 ml of MRS broth media (Liofichem Bacteriology Products) and incubated at 37 °C for 48 h. The single colonies of bacteria were screened for sensitivity to antibiotics, including vancomycin, tetracycline, methicillin, and penicillin, according to the literature^[^^13^^]^.

*Molecular identification of the isolated single colony bacteria*

The genomic DNA of the selected bacterium was extracted in accordance with a previously described protocol^[^^14^^]^, and its molecular identification was confirmed by 16S rDNA gene amplification and direct sequencing^[^^10^^]^. In brief, 1 ml of bacterial suspension was centrifuged at 3000 ×g for 2 min. After removing the supernatant and washing the pellet, 850 µl of buffer (20 mM of NaCl, 100 mM of Tris-HCL, 20 mM of EDTA, and 20% cetyltrimethylammonium bromide, pH 8.0) was added. The suspension was then heated at 65 °C for 1 h and frozen at -70 °C for 30 min; the freeze-thawing cycle was repeated two times. Afterward, 850 µl of chloroform/isoamyl alcohol solution (24:1) was added to the suspension. The samples were subsequently centrifuged at 10000 ×g for 10 min, and 300 µl of the upper phase was mixed with 750 µl of isopropyl alcohol in a separated microtube and incubated at -80 °C overnight. The next day, samples were centrifuged at 10000 ×g for 10 min, and after discarding the supernatant, the pellet of the DNA was left to dry. Finally, 50 µl of sterile distilled water was added to the microtube, and DNA quantity was assessed using Nanodrop OneC (Thermo scientific, USA). Two universal primers (Bioneer, Korea), forward (5'-AGCAGTAGGGAATCTTCCA-3') and reverse (5'-ATTTCACCGCTACACATG-3'), were used to amplify 16S rRNA using the PCR. For each reaction, a 50 μl of reaction mixture containing 25 μl of Master Mix (Eppendorf, USA), 2 μl of each forward and reverse primer, 19 μl of sterile water, and 2 μl of the DNA sample was added to 0.2 ml tube. The amplification program was performed using a denaturing step at 95 °C for 4 min, followed by 32 cycles at 94 °C for 50 seconds, 59 °C for 50 seconds, and 72 °C for 1 min and 40 seconds, with a final 5 min extension at 72 °C. Finally, the PCR products were loaded on a 1.5% agarose gel and visualized by UV illumination. From the four bacterial isolates obtained, one showed the pattern of a clear single band on the 1.5% agarose gel and was further selected for sequencing carried out using the Sanger sequencing method. The 16S rRNA gene sequence was compared with the sequences in the GenBank database by using BLAST (https://blast.ncbi. nlm.nih.gov/Blast.cgi). 

*Cell culture and CM preparation*

Caov-4 cells as the OVC cell line with an epithelial-like morphology were purchased from the National Cell Bank of Iran (Pasteur Institute of Iran, Tehran). The cells were seeded using RPMI-1640 (Sigma-Aldrich, USA) medium supplemented with 10% v/v FBS (Gibco, USA), penicillin-streptomycin (Gibco) in the six-well plates incubating in 95% air and 5% CO_2_ at 37 °C. When the cells reached 80% confluence, the medium was removed and a fresh medium was added without antibiotics. The bacteria were co-cultured with Caov-4 cells by permeable cell culture inserts with a 0.4 µm pore size. After 4 h, the medium was aspirated, centrifuged (3000 ×g, 5 min), and filtered using a 0.22-μm filter. The obtained medium (pH adjusted to 7.2) was defined as the CM. The efficacy of filtration following incubation at 37 °C overnight was confirmed by the lack of bacterial growth upon culture on MRS agar plates. 

*Cell viability assay*

The effect of CM on the viability of Caov-4 cells was determined by a colorimetric assay using MTT (Sigma-Aldrich). Briefly, the cells (1 × 10^4^/well) were seeded in a 96-well plate and cultured in the RPMI-1640 medium (Sigma-Aldrich) containing 10% v/v FBS (Gibco) and penicillin-streptomycin (Gibco) in 95% air and 5% CO_2_ at 37 °C. After 24 h, each well was further treated with different concentrations of CM that comprised co-cultured bacteria with 0.2, 0.5, 1, 1.5, and 2 OD in a medium supplemented with 2% FBS for 12, 24, and 48 h. Then 10 μl of MTT solution (0.01 g in 2 ml PBS) was added to each well and incubated for another 2 h. The culture medium was removed and replaced by 175 μl of dimethyl sulfoxide (Merck, Germany). The experiment was performed in triplicate and also repeated three times. Finally, the absorbance value was measured at 570 nm using a microplate reader (Biotek Instruments, USA). Cell viability was determined using the following equation: percentage of cell viability = (sample OD/control OD) ×100^[^^15^^]^. Since the highest inhibitory effect of CM on Caov-4 cells was observed when OD was 0.5, following 48 h of incubation, we conducted the subsequent experiments on this culture condition.

***DAPI staining and DNA laddering assay***

*DAPI staining method was used to recognize the visual symptoms of apoptosis in cancer cells. The Caov-4 cells were seeded in six-well plates and treated with 0.5 OD of CM for 48 h. At the end of the incubation period, the cells were washed and fixed with 4% paraformaldehyde for 10 min. Then the cells were permeabilized using Triton X-100 (0.1%) for 10 min and washed two times with PBS. The cells were stained with 2 μg/ml of DAPI (Sigma-Aldrich) and incubated in the dark at room temperature for 5 min. Finally, after two times washing, the cells were evaluated using a fluorescent microscope (Olympus system, Japan). The cultured cells were used for DNA laddering assay based on a previously published protocol* ^[^^16^^]^ *. All the experiments were performed in triplicate.*

***Annexin V-PI staining***

*Annexin V-FITC kit was used for the detection of phosphatidylserine externalization and determination of the extent of apoptotic cell death. Briefly, the Caov-4 cells were treated with CM 0.5 OD for 48 h. The samples were prepared according to the kit manufacturer’s protocol. In brief, CM-treated Caov-4 cells were collected and re-suspended in 100 μl of the annexin V binding buffer and 5 μl of annexin V-FITC. After 15 min of incubation in* *the dark conditions at room temperature, the cells were centrifuged (130 ×g, 5 min). Following the removal of the supernatant, 200 μl of the annexin V binding buffer and 5 μl of PI staining solution were added, and the solution was further incubated in darkness at room temperature for 5 min. The cells were analyzed using the FACS Calibur flow cytometry system (Becton Dickinson, San Jose, CA). A total of 10,000 cells per sample were acquired, and the data were analyzed using Cell Quest software (BD Biosciences). *

Gene expression analysis

The Caov-4 cells were treated with 0.5 OD of CM for 12, 24, and 48 h, and then the total RNA was extracted using the TRIzol reagent following the manufacturer's instructions. The 260/280 and 260/230 ratios were assessed using Nanodrop One^C^. RNA (1 µg) from each sample was used for quantitative stem-loop reverse transcription and quantitative real-time PCR analysis. After confirming the appropriate quality and quantity of the extracted total RNA, the corresponding complementary DNA was synthesized using the PrimeScript^TM^ RT reagent kit (TaKaRa, Japan). Then the expression levels of *PTEN*, *BAX*, *BCL2*, *AKT1*, and *GAPDH* (housekeeping) genes were evaluated using appropriate primers listed in Table 1. Quantitative PCR was performed using SYBR Master Mix on a Bio-Rad IQ5 real-time PCR detection system (Hercules, CA, USA). The thermal cycling condition was as follows: 1 cycle at 94 °C for 10 min, followed by 40 cycles at 94 °C for 15 seconds, 60 °C for 30 seconds, and 72 °C for 25 seconds. All reactions were performed in triplicate for each sample and the melting curve was determined at 60-95 °C. The expression levels of each gene were analyzed by Pfaffl method with normalization to the housekeeping gene.

**Table 1:** Sequence of primers used in the study

**Gene**	**Forward primer**	**Reverse primer**
*PTEN*	5'-TCCCAGTCAGTCAGAGGCGCTATG-3'	5'-CACAAACTGAGGATTGCAAGTTC-3'
*BAX*	5'-GATGCGTCCACCAAGAAG-3'	5'-AGTTGAAGTTGCCGTCAG-3'
*BCL2*	5'-GTTCCCTTTCCTTCCATCC-3'	5'-TAGCCAGTCCAGAGGTGAG-3'
*AKT1*	5'-CATCACACCACCTGACCAAT-3'	5'-CTCAAATGCACCCGAGAAAT-3'
*GAPDH*	5'-AAGCTCATTTCCTGGTATGACAACG-3'	5'-TCTTCCTCTTGTGCTCTTGCTGG-3'

***PPI network analysis***

*PPI network analysis was generated based on the medium confidence score of 0.4, and the up- and down-regulated target genes were mapped to the STRING database v10.5 (http://www.string-db.org). The PPI networks were established through all the interaction sources, including text-mining, experiments, databases, co-expression, neighborhood, gene fusion, and co-occurrence.*

***Statis*** t***ical analysis***

*Data were expressed as mean ± SD. Statistical analysis of the data was determined by t-test for two comparisons or ANOVA, followed by the Tukey’s test for multiple comparisons. SPSS software version 21 was used to analyze the data, and a p < 0.05 was considered as statistically significant.*

## RESULTS AND DISCUSSION

***Antibiotic susceptibility of the isolate***

*E. faecium* isolated from vaginal fluid represented great antibacterial susceptibility to the conventional antibiotics used in this study. The bacteria were sensitive to penicillin, methicillin, and tetracycline with 23, 27, and 25 mm diameters of inhibition zone, respectively. However, the bacteria had an intermediate sensitivity to vancomycin with a 17 mm diameter of inhibition zone.

***Effect of CM on cancer cell viability***

**Fig. 1:**
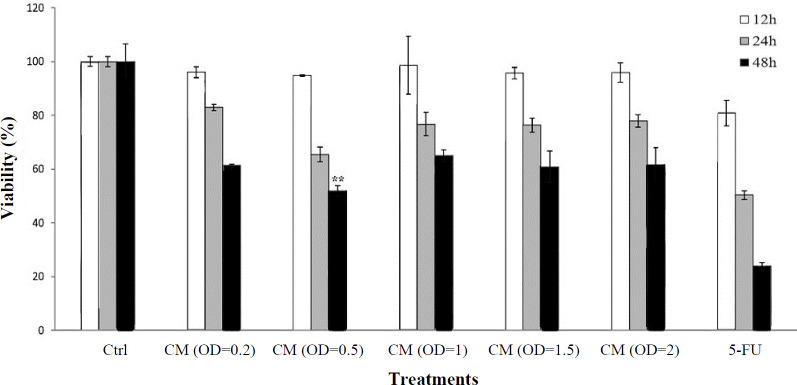
Viability of OVC (Caov-4) cells after treatment with CM of *E. faecium*. Each experiment was conducted in triplicate. 5-FU and the cells without treatment were used as the positive and negative controls, respectively. ^**^Represents statistical significance (*p *< 0.01) compared to other concentrations of CM with different incubation times.

To find the optimum concentration of CM and proper incubation time for inhibiting Caov-4 cell growth, we cultured the OVC cells with varying concentrations of CM that comprised co-cultured bacteria with 0.2, 0.5, 1, 1.5, and 2 OD, as well as with the 5-FU at 100 µg/ml as the positive control for 12, 24, and 48 h. Our results showed that CM could reduce the viability of cancer cells at all the concentrations used at three incubation times (Fig. 1); however, the inhibitory effect was higher when the incubation time increased to 48 h. Moreover, we found the most inhibitory effect when the cells were treated with CM at 0.5 OD for 48 h (percentage of living cells: 51.77 ± 1.97%**).** In accordance with our results, Thirabunyanon* and *Hongwittayakorn^[15]^ have reported that the cell-free supernatant of enterococcus could inhibit the growth of cancer cells, and *E. faecium* FP51 isolated from the newborn feces has anticancer activity against Caco-2 cells. Chimchang et al. ^[17]^ have also demonstrated that *E. faecium*-derived CM display*s* antiproliferative activity against human monocytic leukemic cells (U937).

***Effect of CM on the apoptosis of cancer cell***

**Fig. 2:**
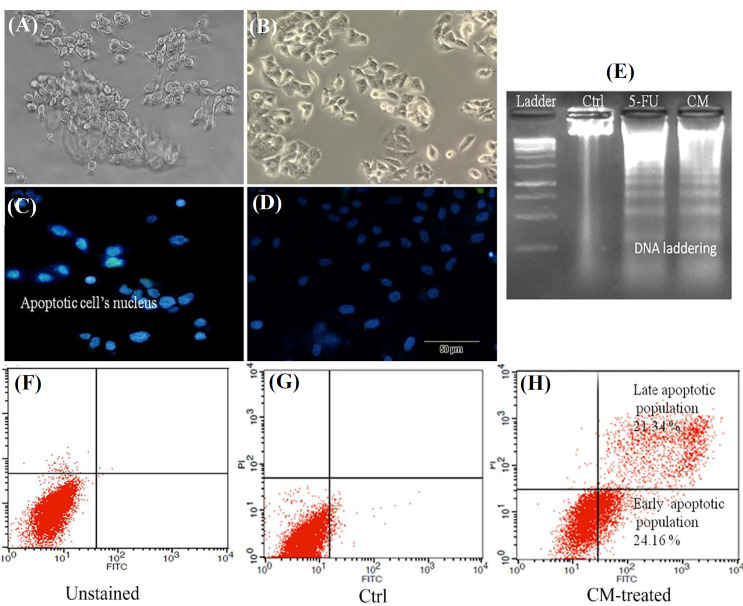
Apoptosis of OVC (Caov-4) cells after treatment with CM of *E. faecium*. (A and B) the light microscopic monitoring of CM-treated and control (Ctrl) cells, respectively; (C and D) nucleus fragmentation using DAPI staining in CM-treated and control cells, respectively; (E) DNA laddering assay in control (Ctrl), 5-FU treated, and CM treated (0.5 OD) cells; (F-H) unstained, untreated, and CM-treated cells (0.5 OD), respectively, following Annexin V-PI staining; 45% of apoptotic cells were observed in the CM-treated group.

*After DAPI staining, the Caov-4 cells were morphologically evaluated, and cell membrane blebbing and chromatin condensation were considered as the hallmarks of apoptotic cells. Our results confirmed that CM could induce apoptosis in OVC cells when incubated for 48 hours (* Fig. 2A-2D *). As shown in* Figure 2C *, the Caov-4 cells had a fragmented nucleus following treatment with CM at 0.5 OD for 48 h. DNA laddering assay also confirmed higher DNA fragmentation in Caov-4 cells treated with CM 0.5 OD for 48 h, as compared to the untreated cells (* Fig. 2E *). Moreover, Annexin V-PI staining indicated a significantly increased population of apoptotic cells (about 45%) in the cell group treated with CM compared to the untreated cells (* Fig. 2F-2H *). In accordance with our* *findings, a previous study has also represented the apoptotic effect of probiotics*^[^^18^^]^*. In this context, apoptosis-related morphological changes have been reported upon the treatment of cancer cells with probiotics*^[^^19^^,^^20^^]^*. It has also been shown that the isolated strains of *E. faecalis* could induce apoptosis in MC3T3 osteoblasts*^[^^21^^]^*.* *The results indicated that the new strain of SP-OV14 (accession number: MT555786) had 100% homology with *E. faecium*.*

**Fig. 3:**
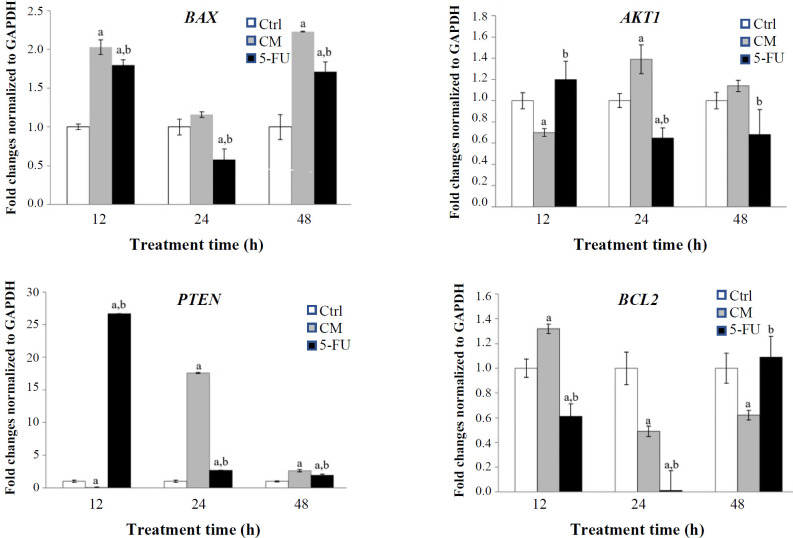
Expression of *BAX*, *PTEN*, *AKT1*, and *BCL2* genes in Caov-4 cells treated with CM derived from *E. faecium* (0.5 OD) for 12, 24, and 48 h. A significant difference (*p* < 0.05) is presented in comparison with ^a ^untreated (Ctrl: control) and ^b ^CM-treated groups.

***Gene expression of pro- and antiapoptotic genes following treatment with CM***

The results showed that treatment of the Caov-4 cells with *E. faecium*-derived CM (0.5 OD) significantly upregulated proapoptotic gene, *BAX*. Treatment of the cells with 5-FU showed similar effects; however, the CM induced *BAX* expression more than that of 5-FU when the incubation time was 48 h. The expression of *PTEN* as another proapoptotic gene, also increased following treatment with CM or 5-FU. However, the expression of *PTEN *decreased after the CM treatment within a short duration of 12 hours. For *AKT1*, an antiapoptotic gene, CM exhibited a significant decrease and increase in its expression after 12 and 24 hours of incubation, respectively. The antiapoptotic gene *BCL2 *also exhibited a decrease in expression subsequent to the administration of CM for 24 and 48 hours. Notably, the degree of gene downregulation was observed to exceed that of 5-FU at 48 hours. After 12 h of incubation, the CM did not show any inhibitory effect on the *BCL2* expression. (Fig. 3). These results were consistent with previous studies reporting the positive effects of probiotics on tumor suppressor genes such as *PTEN* and *BAX *^[^^22^^-^^24^^]^. Nonetheless, our findings indicate that the gene expression was more significantly impacted by 5-FU than CM during the initial treatment period. This observation may be attributed to the mode of action of 5-FU, which acts through the direct suppression of thymidylate synthase and consequently the replication of DNA^[^^25^^]^. While the CM may act through different direct and indirect mechanisms including affecting growth- and apoptosis-related signaling pathways and gene transcription, further studies are required to discover the active substances in CM and the underlying mechanism in cancer cells. In our study, *E. faecium *CM could downregulate *BCL2* as an antiapoptotic gene, especially when the incubation time was 48 h. De Leblanc et al.^[^^26^^]^ have also reported that the expression of *BCL2* decreased when probiotic *Lactobacillus helveticus* R389 was used in the mammary glands. The current investigation has demonstrated no noteworthy impact of CM on *AKT* expression, a finding which has been substantiated by a recent research conducted by Lin et al.^[^^27^^]^. Considering the impact of CM on the expression of *PTEN* and *BAX* and given that the CAOV-4 cell line carries a loss-of-function mutation in the p53 gene, we proceeded to assess other functional hub signaling pathways. According to our predicted network, Bacteria activated caspases induce apoptosis in cancerous cells.

**Fig. 4:**
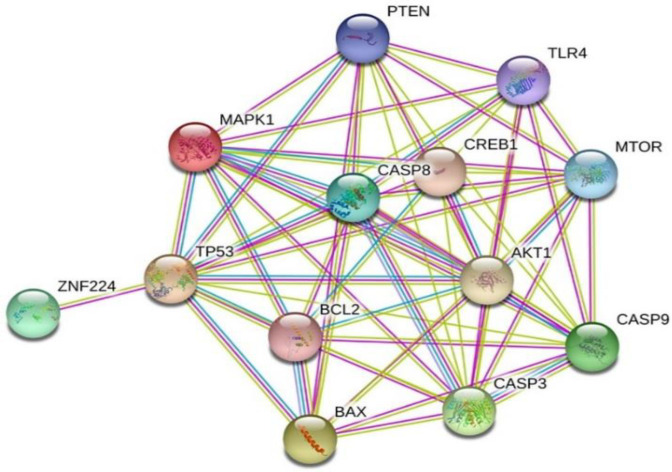
PPI network between pro-and antia

We have performed an assessment of the PPI network that exists between pro and antiapoptotic factors in conjunction with TP53 and caspase signal transduction. To accomplish this analysis, we utilized the STRING Database v. 10.5. Our aim was to provide clarity on the potential mechanism of the anticancer effect of CM (Fig. 4). CAOV-4 cell line harbors a loss-of-function mutation in the p53 gene and is resistance to the apoptosis^[^^28^^]^. In the current bioinformatics study, we showed that bacteria induced the hub signaling pathway involved in the apoptosis of cancerous cells. According to the PPI network, the expression of casp8 and casp9 was found to be upregulated in the treated cells. This upregulation induces apoptosis, even in the cells that harbor a loss-of-function mutation in the p53 gene and are resistant to apoptosis. It is notable that stronger associations are represented by thicker lines in this network. The PPI network was predicted using medium confidence scores with a value of 0.40. Additionally, the MCL clustering algorithm with an inflation parameter of 5 was used to cluster the network. 

## Conclusion

Our findings demonstrate that the CM of human-derived *E. faecium *have anticancer effects on OVC cells in a concentration- and time-dependent manner, and the anticancer effects of the CM are likely through the induction of apoptosis and suppression of antiapoptotic genes. It could be suggested that probiotics have the potential to prevent the development of cancer not only by inhibiting the growth of pathogenic bacteria but also by producing anticancer agents and exerting direct influence on neoplastic cells. Therefore, administration of probiotics or their CM can be considered a potential therapeutic strategy for cancer. 

## DECLARATIONS

### Acknowledgments

We would like to thank the personnel of Stem Cell and Regenerative Medicine Institute and Laboratory of Pharmaceutics of Tabriz University of Medical Sciences (Tabriz, Iran) for providing laboratory facilities and workspaces.

### Ethical statement

The study protocol was approved by the Ethics Committee of the Tabriz University of Medical Sciences, Tabriz, Iran (ethical code: IR.TBZMED.REC.1396.1283). Informed consents were obtained from all the participants.

### Data availability

The data sets that were analyzed in the course of this study are accessible upon reasonable request from the corresponding author.

### Author contributions

SP: data gathering and writing-original draft; AFK: manuscript scanning and revision of the manuscript; AB: conceptualization and supervision of lab work; SB: biochemical and statistical analysis; SB: revision of the manuscript; AF: conception of the original idea and the subsequent study design; MS: supervision of the project. All authors have read and approved the final version of the manuscript.

### Conflict of interest

None declared.

### Funding/support

This study was financially supported by Vice Chancellor for Research and Technology, Tabriz University of Medical Science, Tabriz, Iran.
